# Causal inference between aggressive extrathyroidal extension and survival in papillary thyroid cancer: a propensity score matching and weighting analysis

**DOI:** 10.3389/fendo.2023.1149826

**Published:** 2023-05-24

**Authors:** Ming Xu, Zihan Xi, Qiuyang Zhao, Wen Yang, Jie Tan, Pengfei Yi, Jun Zhou, Tao Huang

**Affiliations:** Department of Breast and Thyroid Surgery, Union Hospital, Tongji Medical College, Huazhong University of Science and Technology, Wuhan, China

**Keywords:** papillary thyroid cancer, extrathyroidal extension, survival analysis, Surveillance, Epidemiology, and End Results (SEER), causal inference analysis

## Abstract

**Background:**

Extrathyroidal extension is a major risk factor for poor prognosis in papillary thyroid cancer. However, the effect of different degrees of extrathyroidal extension on prognosis remains controversial. We performed a retrospective study to elucidate how the extent of extrathyroidal extension in papillary thyroid cancer affected the clinical prognosis of patients and its covariates.

**Methods:**

The study included 108,426 patients with papillary thyroid cancer. We categorized the extent of extension into none, capsule, strap muscles, and other organs. Three causal inference methods for retrospective studies, namely, inverse probability of treatment weighting, standardized mortality ratio weighting, and propensity score matching analysis, were used to minimize potential selection bias. Kaplan–Meier analysis and univariate Cox regression analyses were applied to analyze the precise effect of ETE on survival in papillary thyroid cancer patients.

**Results:**

In the Kaplan–Meier survival analysis, only extrathyroidal extension into or beyond the strap muscles was statistically significant for both overall survival (OS) and thyroid cancer-specific survival (TCSS). In univariate Cox regression analyses before and after matching or weighting based on causal inference, extrathyroidal extension into soft tissues or other organs is a high-risk factor for both overall survival and thyroid cancer-specific survival. Sensitivity analysis revealed that lower overall survival was observed in patients with older age (≥55) and larger tumor size (>2 cm) of papillary thyroid cancer with extrathyroidal extension into or beyond the strap muscles.

**Conclusions:**

Our study indicates that extrathyroidal extension into soft tissues or other organs is a high-risk factor in all papillary thyroid cancer. Even though invasion into the strap muscles did not seem to be a marker for poor prognosis, it still impaired the overall survival of patients with older age (≥55 years old) or larger tumor size (>2 cm). Further investigation is needed to confirm our results and to clarify further risk factors independent of extrathyroidal extension.

## Introduction

In papillary thyroid cancer (PTC), extrathyroidal extension (ETE) is an important risk factor for poor prognosis (1–7). ETE is defined as the primary tumor invading adjacent tissues beyond the thyroid gland (8–10). ETE has been one of the most important and controversial determinants of T stage in the AJCC staging system. In the seventh edition of AJCC staging, defined as microscopic tumor extension into skeletal muscles (i.e., strap muscles) or perithyroidal soft tissues (10), minimal ETE was considered a risk factor for prognosis. Patients with minimal ETE were classified as T3 and stage III. Gross ETE was categorized as T4 and stage IVa or IVb. These standards were revised in the eighth edition of the AJCC staging system (11–14). Minimal ETE has been removed from the staging system, demonstrating its limited impact on prognosis. Gross extension into the strap muscles only is considered T3b and stage II, while ETE into the perithyroidal soft tissues is ambiguous in the eighth edition (15). Further macroscopic extension into subcutaneous soft tissues or other organs is still marked as T4, but the stage is slightly downgraded to stage III or IVa. The change in the staging system will affect the extent of surgical resection and the decision of radioactive iodine (RAI) therapy (16, 17). Therefore, debates on the relationship between ETE and patient prognosis are necessary. It is accepted that ETE is associated with an increased risk of recurrence and death in PTC patients (3, 18–21). An analysis of more than 200,000 patients with differentiated thyroid cancer in the National Cancer Database has demonstrated that both minimal and extensive ETE are associated with adverse prognostic impact, while minimal ETE has a smaller hazard ratio (22). However, most of the studies have shown that minimal ETE does not significantly affect clinical outcomes (23–28). Gross invasion into the strap muscles and perithyroidal tissues also showed a variable influence on disease recurrence and outcome (4, 22, 29–31). Therefore, the extent of ETE still needs further investigation to help us classify patient prognosis and guide patient treatment.

Causal inference from observational studies has been a research difficulty due to the presence of covariates. In papillary thyroid cancer, extrathyroidal extension is an essential feature of poor prognosis. Researchers have explored how its extent influences patient recurrence, but extrathyroidal extension coexists with other features such as larger tumor size, multifocality, lymph node metastasis (32), etc. Their coexistence has been a confounding factor in the causal inference analysis of extrathyroidal extension. Therefore, we conducted various matching and weighting methods to investigate the true effect of ETE in papillary thyroid cancer.

In this retrospective study, we used clinical data from the Surveillance, Epidemiology, and End Results (SEER) program to investigate the effect of the extent of ETE on the survival of PTC patients. Multiple matching and weighting methods were used to control for potential covariates in the observational study to provide robust evidence for our findings. We attempted to elucidate how ETE affects patient survival and provide supportive evidence for the revision of the AJCC eighth edition on tumor staging.

## Methods

### Data source and selection

The National Cancer Institute’s SEER program is one of the largest open cancer databases in the world (33). This retrospective study was based on data collected using SEER*Stat version 8.3.5 (http://seer.cancer.gov/seerstat/). We extracted patients diagnosed with PTC (8050/3, 8260/3, 8340/3, 8341/3, 8342/3, 8343/3, 8344/3) using the International Classification of Diseases for Oncology, Third Revision (ICD-O-3). Informed consent was not required for our work because the database is publicly available.

Patients with PTC were included according to the following inclusion criteria: 1) patients diagnosed with papillary thyroid carcinoma between 2004 and 2018, during which the extent of ETE is available; 2) papillary thyroid carcinoma was the first diagnosed primary tumor; 3) patients were followed up at least once and survival time was recorded; and 4) all covariates of interest were known and available. Patients who underwent non-specific surgery of the primary tumor or only lymph node biopsy were excluded.

### Variables and outcomes

The patient characteristics we focused on were age at diagnosis, gender, tumor size, multifocality, extension, lymph node status, metastasis status, and surgical treatment including the primary tumor and lymph nodes. Age at diagnosis was divided into three groups: younger than 20 years, 20 to 54 years, and 55 years or older. Gender was dichotomized into male and female. Tumor size was classified into four groups, namely, ≤1 cm, >1 and ≤2 cm, >2 and ≤4 cm, and >4 cm, using the CS tumor size code. Multifocality was classified as solitary (code 10) or multifocal (code 20) according to CS site-specific factor 1 (solitary *vs*. multifocal tumor). Extension was classified as no extrathyroidal extension (no ETE) and extrathyroidal extension (ETE). ETE was further specified as ETE into capsule, strap muscles, soft tissues, and other organs including parathyroid gland, nerves, cartilage, sternocleidomastoid muscle, esophagus, larynx, trachea, blood vessels, and other extension into mediastinal tissues or prevertebral fascia according to the CS extension code. Lymph node status was reported as negative (N0) if no positive lymph nodes were found or positive (N1) if positive lymph nodes were found and reported in the CS lymph node code. Metastasis status was labeled as none, distant positive lymph nodes, or distant metastasis according to the CS Mets at DX code. Surgery on the primary tumor was divided into four categories, namely, no surgery, partial surgery, lobectomy, and near total/total thyroidectomy, as retrieved by the RX Summ–Surg Prim Site code. The lymph node surgery was marked as none or partial dissection as indicated by the RX Summ–Scope Reg LN Sur code. In our study, survival was defined as the time from diagnosis to the end event or last follow-up. Patients who were alive at the last follow-up were considered as right censored in the survival analysis.

### Propensity scoring matching and weighting

The propensity scores, as formalized, are used to define the tendency of a patient to receive a certain treatment or characteristic, e.g., in our research, tumor extension. We calculated propensity scores using logistic regression (34). The confounders of our interest are age, gender, tumor size, multifocality, lymph node status, metastatic status, extent of surgery at the primary site, and central cervical lymph nodes. Using propensity scores, we performed propensity score matching (PSM) to reduce the influence of other covariates. Pairs of patients with or without ETE were matched using the “MatchIt” package in R. The matching method we used was the nearest method with a caliper width of 0.01 and a ratio of 1:1 without replacement. We assessed balance using standardized mean differences (SMDs) as previously reported (35).

In addition to matching, we considered alternative propensity adjustment approaches, such as inverse probability of treatment weighting (IPTW) and standardized mortality ratio weighting (SMRW). Unlike matching, these methods preserve the original sample characteristics without reducing the sample size. In IPTW, the entire population is taken as the reference population. We used stabilized weights in IPTW (36, 37) as follows:


We=Pt/PS

for patients with extension, and


Wc=(1−Pt)/(1−PS)

for the control population, where PS is the propensity score of an individual and Pt is the proportion of patients with extension in the whole population. On the contrary, in SMRW, treated patients are given a weight of 1, which means that the treated population is considered the standard population. The stabilized weights (38) we used for SMRW are as follows:


We=1

and


Wc=[PS(1−Pt)/(1−PS)Pt],

where PS is the propensity score of an individual and Pt is the proportion of patients with extension in the whole population.

### Statistical analysis

Clinicopathologic factors were compared by chi-squared test (*χ* ^2^). Survival curves were obtained by Kaplan–Meier analysis, and significance was tested by the log-rank test, except for the analysis after PSM, because the stratified log-rank test was used. Univariate Cox proportional hazards models were performed to analyze hazard ratios (HRs) and 95% confidence intervals (95% CIs) for different degrees of tumor extension. All analyses were performed using the survival and survminer packages in R (version 3.6.0). All *p*-values and 95% CIs were tested using two-tailed tests, and *p<*0.05 was considered statistically significant.

## Results

### Patients with ETE have aggressive characteristics

**Figure 1:**
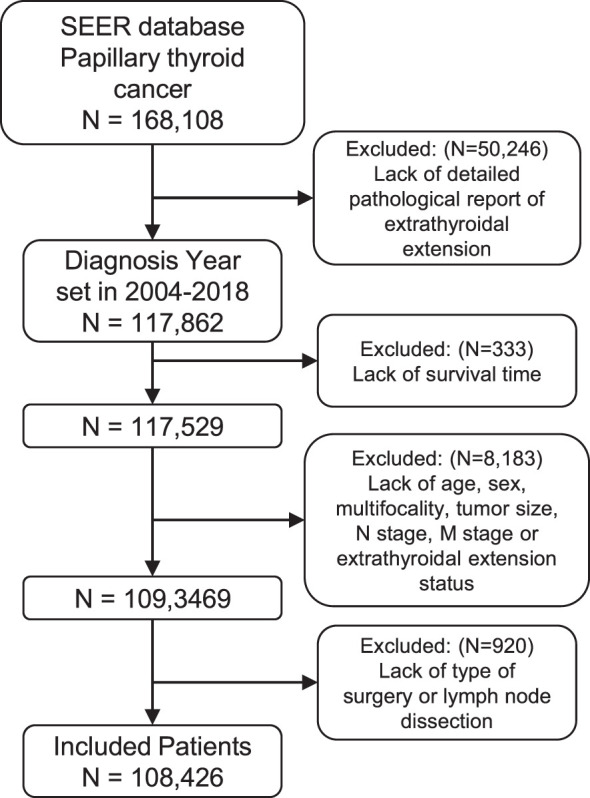
Flow diagram of patient selection for the study cohort. ICD-O-3 histology codes as follows: 8050/3 (papillary carcinoma), 8260/3 (papillary adenocarcinoma), 8340/3 (papillary carcinoma, follicular variant), 8341/3 (papillary microcarcinoma), 8342/3 (papillary carcinoma, oxyphilic cell), 8343/3 (papillary carcinoma, encapsulated), and 8344/3 (papillary carcinoma, columnar cell). SEER, Surveillance, Epidemiology, and End Results.

We identified 168,108 patients with PTC from the SEER database. According to the inclusion criteria, 108,426 patients were included in this study (**Figure 1**), with a median (range) follow-up of 53 months (0–143).

The patients were divided into two groups: PTC with ETE (*n* = 25,302, 23.3%) and PTC without ETE (*n* = 83,124, 76.6%). Patients with ETE were divided into four groups according to the extent of ETE: ETE in capsule, ETE in strap muscles, ETE in soft tissues, and ETE in other organs. The clinicopathologic characteristics are shown in **Table 1**. The results showed that patients with ETE had risk factors including older age, male over female, multifocality, larger tumor size, lymph node metastasis, distant metastasis, and more aggressive surgical treatment.

**Table 1:** Clinical characteristics of PTC patients with no extrathyroidal extension *versus* with extrathyroidal extensions.

Characteristics	No ETE	ETE					*p*-value*
		All ETE	Capsule	Strap muscles	Soft tissue	Other organs	
	*n* = 83,124	*n* = 25,302	*n* = 6,992	*n* = 8,196	*n* = 6,681	*n* = 3,433	
**Age at diagnosis**							<0.001
≤19 (%)	1,323 (1.6)	605 (2.4)	153 (2.2)	209 (2.6)	176 (2.6)	67 (2.0)	
20–54 (%)	51,539 (62.0)	14,756 (58.3)	4,492 (64.2)	4,830 (58.9)	3,948 (59.1)	1,486 (43.3)	
≥55 (%)	30,262 (36.4)	9,941 (39.3)	2,347 (33.6)	3,157 (38.5)	2,557 (38.3)	1,880 (54.8)	
**Sex**							<0.001
Female (%)	65,541 (78.8)	18,380 (72.6)	5,347 (76.5)	5,990 (73.1)	4,755 (71.2)	2,288 (66.6)	
Male (%)	17,583 (21.2)	6,922 (27.4)	1,645 (23.5)	2,206 (26.9)	1,926 (28.8)	1,145 (33.4)	
**Multifocality**							<0.001
Solitary (%)	50,998 (61.4)	11,918 (47.1)	3,772 (53.9)	3,575 (43.6)	2,906 (43.5)	1,665 (48.5)	
Multifocal (%)	32,126 (38.6)	13,384 (52.9)	3,220 (46.1)	4,621 (56.4)	3,775 (56.5)	1,768 (51.5)	
**Tumor size**							<0.001
≤1 cm (%)	41,326 (49.7)	4,375 (17.3)	1,579 (22.6)	1,437 (17.5)	1,098 (16.4)	261 (7.6)	
≤2 cm (%)	22,785 (27.4)	8,809 (34.8)	2,257 (32.3)	3,147 (38.4)	2,520 (37.7)	885 (25.8)	
≤4 cm (%)	14,673 (17.7)	8,284 (32.7)	2,205 (31.5)	2,638 (32.2)	2,131 (31.9)	1,310 (38.2)	
>4 cm (%)	4,340 (5.2)	3,834 (15.2)	951 (13.6)	974 (11.9)	932 (14.0)	977 (28.5)	
**N category**							<0.001
N0 (%)	69,877 (84.1)	13,524 (53.5)	5,353 (76.6)	3,907 (47.7)	3,058 (45.8)	1,206 (35.1)	
N1 (%)	13,247 (15.9)	11,778 (46.5)	1,639 (23.4)	4,289 (52.3)	3,623 (54.2)	2,227 (64.9)	
**Metastasis**							<0.001
None (%)	82,795 (99.6)	24,623 (97.3)	6,942 (99.3)	8,061 (98.4)	6,546 (98.0)	3,074 (89.5)	
Distant LN (%)	41 (0.0)	59 (0.2)	2 (0.0)	12 (0.1)	17 (0.3)	28 (0.8)	
Distant metastasis (%)	288 (0.3)	620 (2.5)	48 (0.7)	123 (1.5)	118 (1.8)	331 (9.6)	
**Surgery**							<0.001
Near TT/TT (%)	69,267 (83.3)	23,866 (94.3)	6,316 (90.3)	7,936 (96.8)	6,434 (96.3)	3,180 (92.6)	
Lobectomy (%)	12,052 (14.5)	1,206 (4.8)	640 (9.2)	232 (2.8)	226 (3.4)	108 (3.1)	
Partial (%)	686 (0.8)	105 (0.4)	29 (0.4)	21 (0.3)	14 (0.2)	41 (1.2)	
No surgery (%)	1,119 (1.3)	125 (0.5)	7 (0.1)	7 (0.1)	7 (0.1)	104 (3.0)	
**LN surgery**							<0.001
None (%)	44,177 (53.1)	7,869 (31.1)	3,194 (45.7)	2,099 (25.6)	1,734 (26.0)	842 (24.5)	
Partial (%)	38,947 (46.9)	17,433 (68.9)	3,798 (54.3)	6,097 (74.4)	4,947 (74.0)	2,591 (75.5)	

p-value* indicated the significance between no extrathyroidal extension and all extrathyroidal extensions.

PTC, papillary thyroid cancer; ETE, extrathyroidal extension; LN, lymph node; TT, total thyroidectomy; SMD, standardized mean difference.

Because patients with ETE tended to have more invasive characteristics than those without, we performed additional analyses using three propensity adjustment approaches, namely, PSM, IPTW, and SMRW, to adjust for covariate factors. The SMD was calculated to assess the validity of the adjustment and weighting (**Supplementary Figure 1**). SMD<0.1 was considered to achieve a covariate balance between no ETE groups and ETE groups (34). After weighting, all covariates are balanced as shown in **Table 2** and **Supplementary Figure 1**. The results showed that the three approaches tended to be consistent, further confirming the accuracy of these methods in reducing the influence of covariates.

**Table 2:** Propensity-matched clinical characteristics of PTC patients with no extrathyroidal extension *versus* with extrathyroidal extensions.

Characteristics	All data		SMD	After IPTW		SMD	After SMRW		SMD	After PSM		SMD
	No ETE	ETE		No ETE	ETE		No ETE	ETE		No ETE	ETE	
	*n* = 83,124	*n* = 25,302		*n* = 83,142	*n* = 24,953		*n* = 83,199	*n* = 25,302		*n* = 22,904	*n* = 22,904	
**Age at diagnosis**			0.195			0.04			0.024			0.011
≤19 (%)	1,323 (1.6)	605 (2.4)		1,476 (1.8)	447 (1.8)		1,979 (2.4)	605 (2.4)		535 (2.3)	507 (2.2)	
20–54 (%)	51,539 (62.0)	14,756 (58.3)		51,065 (61.4)	15,804 (63.3)		49,508 (59.5)	14,756 (58.3)		13,972 (61.0)	14,069 (61.4)	
≥55 (%)	30,262 (36.4)	9,941 (39.3)		30,601 (36.8)	8,702 (34.9)		31,712 (38.1)	9,941 (39.3)		8,397 (36.7)	8,328 (36.4)	
**Sex**			0.135			0.007			0.008			0.001
Female (%)	65,541 (78.8)	18,380 (72.6)		64,423 (77.5)	19,406 (77.8)		60,749 (73.0)	18,380 (72.6)		17,077 (74.6)	17,083 (74.6)	
Male (%)	17,583 (21.2)	6,922 (27.4)		18,719 (22.5)	5,547 (22.2)		22,450 (27.0)	6,922 (27.4)		5,827 (25.4)	5,821 (25.4)	
**Multifocality**			0.186			0.038			0.018			0.002
Solitary (%)	50,998 (61.4)	11,918 (47.1)		48,072 (57.8)	13,956 (55.9)		38,458 (46.2)	11,918 (47.1)		11,030 (48.2)	11,011 (48.1)	
Multifocal (%)	32,126 (38.6)	13,384 (52.9)		35,070 (42.2)	10,997 (44.1)		44,741 (53.8)	13,384 (52.9)		11,874 (51.8)	11,893 (51.9)	
**Tumor size**			0.535			0.026			0.011			0.007
≤1 cm (%)	41,326 (49.7)	4,375 (17.3)		35011 (42.1)	10,195 (40.9)		14,266 (17.1)	4,375 (17.3)		4,340 (18.9)	4,366 (19.1)	
≤2 cm (%)	22,785 (27.4)	8,809 (34.8)		24323 (29.3)	7,481 (30.0)		29,376 (35.3)	8,809 (34.8)		8,602 (37.6)	8,598 (37.5)	
≤4 cm (%)	14,673 (17.7)	8,284 (32.7)		17577 (21.1)	5,346 (21.4)		27,115 (32.6)	8,284 (32.7)		7,247 (31.6)	7,187 (31.4)	
>4 cm (%)	4,340 (5.2)	3,834 (15.2)		6231 (7.5)	1,931 (7.7)		12,442 (15.0)	3,834 (15.2)		2,715 (11.9)	2,753 (12.0)	
**N category**			0.577			0.014			0.001			0.006
N0 (%)	69,877 (84.1)	13,524 (53.5)		63,953 (76.9)	19,041 (76.3)		44,491 (53.5)	13,524 (53.5)		13,532 (59.1)	13,462 (58.8)	
N1 (%)	13,247 (15.9)	11,778 (46.5)		19,189 (23.1)	5,912 (23.7)		38,708 (46.5)	11,778 (46.5)		9,372 (40.9)	9,442 (41.2)	
**Metastasis**			0.218			0.004			0.006			0.011
None (%)	82,795 (99.6)	24,623 (97.3)		82,374 (99.1)	24,714 (99.0)		80,989 (97.3)	24,623 (97.3)		22,636 (98.8)	22,655 (98.9)	
Distant LN (%)	41 (0.0)	59 (0.2)		81 (0.1)	24 (0.1)		213 (0.3)	59 (0.2)		34 (0.1)	26 (0.1)	
Distant metastasis (%)	288 (0.3)	620 (2.5)		687 (0.8)	215 (0.9)		1,997 (2.4)	620 (2.5)		234 (1.0)	223 (1.0)	
**Surgery**			0.3			0.057			0.015			0.012
Near TT/TT (%)	69,267 (83.3)	23,866 (94.3)		71,469 (86.0)	21,871 (87.6)		78,705 (94.6)	23,866 (94.3)		21,553 (94.1)	21,515 (93.9)	
Lobectomy (%)	12,052 (14.5)	1,206 (4.8)		10,134 (12.2)	2,751 (11.0)		3,831 (4.6)	1,206 (4.8)		1,167 (5.1)	1,182 (5.2)	
Partial (%)	686 (0.8)	105 (0.4)		595 (0.7)	139 (0.6)		294 (0.4)	105 (0.4)		89 (0.4)	95 (0.4)	
No surgery (%)	1,119 (1.3)	125 (0.5)		944 (1.1)	192 (0.8)		369 (0.4)	125 (0.5)		95 (0.4)	112 (0.5)	
**LN surgery**			0.33			0.028			0.001			0.005
None (%)	44,177 (53.1)	7,869 (31.1)		39,896 (48.0)	11,624 (46.6)		25,829 (31.0)	7,869 (31.1)		7,845 (34.3)	7,796 (34.0)	
Partial (%)	38,947 (46.9)	17,433 (68.9)		43,246 (52.0)	13,329 (53.4)		57,370 (69.0)	17,433 (68.9)		15,059 (65.7)	15,108 (66.0)	

SMD assessed the balance between no extrathyroidal extension and all extrathyroidal extensions.

PTC, papillary thyroid cancer; ETE, extrathyroidal extension; LN, lymph node; TT, total thyroidectomy; SMD, standardized mean difference.

### Extension into the strap muscles, soft tissues, and other organs worsens patient’s survival in Kaplan–Meier analysis

**Figure 2:**
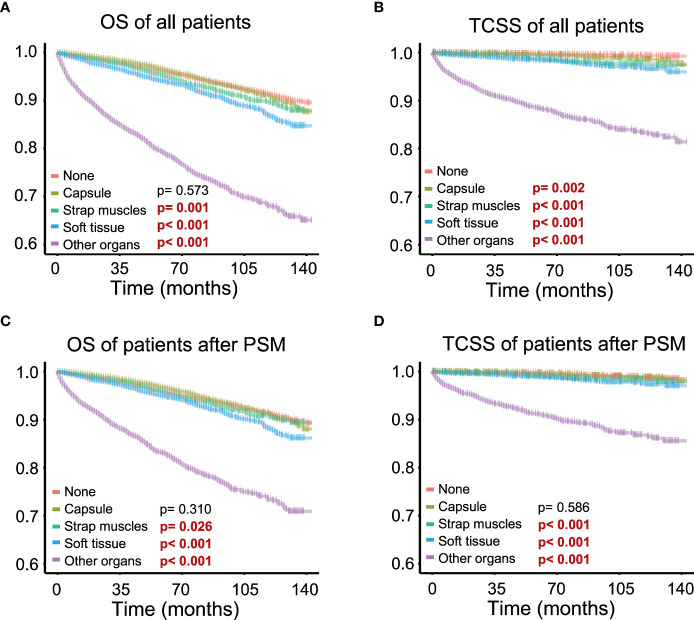
Different extents of extrathyroidal extension and survival; survival probability ranges from 0.6 to 1.0. **(A)** Extrathyroidal extension (ETE) into the strap muscles, soft tissues, and other organs all significantly impaired overall survival in all patients. **(B)** ETE into the strap muscles, soft tissues, and other organs all significantly impaired thyroid cancer-specific survival in all patients. **(C)** ETE into the strap muscles, soft tissues, and other organs all significantly impaired overall survival in patients after PSM. **(D)** ETE into the strap muscles, soft tissues, and other organs all significantly impaired thyroid cancer-specific survival in patients after PSM.

Kaplan–Meier survival analysis was performed to evaluate the prognostic value of different degrees of ETE in PTC patients. The OS rate of patients with ETE in the strap muscles, soft tissues, and other organs was significantly worse than that of patients without ETE (*p* = 0.001, *p*< 0.001, *p*< 0.001, *p*< 0.001), but there was no significant change in patients with ETE in the capsule (*p* = 0.573) (**Figure 2A**). Therefore, ETE into or beyond the strap muscles is a risk factor for the OS rate of thyroid cancer. At the same time, the thyroid cancer-specific survival rate of patients in the other four groups was worse than that of patients without ETE (*p* = 0.002, *p*< 0.001, *p*< 0.001, *p*< 0.001) (**Figure 2B**).

Survival analysis of patients after PSM showed that patients with ETE in the strap muscles and patients with ETE in soft tissues and other organs had a worse prognosis than patients without ETE in both OS (*p* = 0.026, *p*< 0.001, *p*< 0.001) and TCSS (*p*< 0.001, *p*< 0.001, *p*< 0.001, *p*< 0.001, **Figures 2C, D**). Capsular invasion was not a significant factor for patient survival in TCSS after PSM (*p* = 0.002 and *p* = 0.586 before and after PSM, respectively). These results further indicated that ETE into or beyond the strap muscles was a poor prognostic factor for both OS and TCSS using Kaplan–Meier survival analysis.

### Invasion into the strap muscles shows varied effects on patients’ overall and specific prognosis in univariate Cox regression analyses

**Figure 3:**
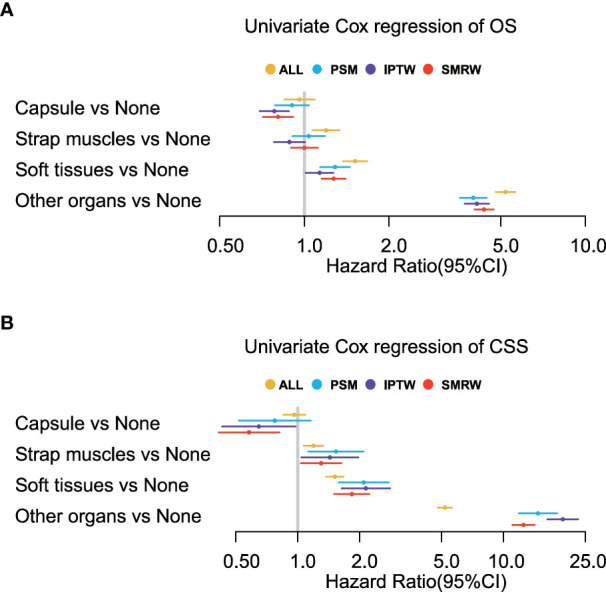
Univariate Cox regression analyses of **(A)** overall survival (OS) and **(B)** thyroid cancer-specific survival (TCSS) for the extension variables. Extension into the strap muscles is not a risk factor in OS but in TCSS.

We performed univariate Cox regression analysis on the four groups of patients, namely, all patients, filtered patients by PSM, weighted patients by IPTW, and weighted patients by SMRW, to calculate the hazard ratios. As shown in **Figure 3**, compared with patients without ETE, ETE in the capsule was not a risk factor for both OS (HR = 0.90, 95% CI = 0.79–1.04, *p* = 0.153, in patients after PSM; HR = 0.78, 95% CI = 0.69–0.88, *p* = 5.564, in patients after IPTW; HR = 0.80, 95% CI = 0.71–0.91, *p*< 0.001, in patients after SMRW) and TCSS (HR = 0.77, 95% CI = 0.52–1.16, *p* = 0.211, in patients after PSM; HR = 0.65, 95% CI = 0.43–0.98, *p* = 0.038, in patients after IPTW; HR = 0.58, 95% CI = 0.41–0.81, *p* = 0.002, in patients after SMRW). Similarly, ETE in the strap muscles was not a risk factor for OS (HR = 1.04, 95% CI = 0.91–1.18, *p* = 0.595, in patients after PSM; HR = 0.89, 95% CI = 0.78–1.00, *p* = 0.059, in patients after IPTW; HR = 1.00, 95% CI = 0.90–1.12, *p* = 0.998, in patients after SMRW). Compared with non-parametric analysis, Cox regression gives specific hazard ratios in the survival analysis. Therefore, we believe that strap muscle invasion was not a predictor of overall prognosis. However, ETE into the strap muscles was a high-risk factor in TCSS (HR = 1.53, 95% CI = 1.13–2.09, *p* = 0.007, in patients after PSM; HR = 1.43, 95% CI = 1.04–1.97, *p* = 0.027, in patients after IPTW; HR = 1.30, 95% CI = 1.04–1.63, *p* = 0.024, in patients after SMRW). ETE to soft tissues and ETE to other organs were always poor prognostic factors for both OS (HR > 1, *p*< 0.050) and TCSS (HR > 1, *p*< 0.050).

### Extension into the strap muscles only affects patients’ outcomes in elderly age (≥55 years old) and larger tumor size (>2 cm)

**Figure 4:**
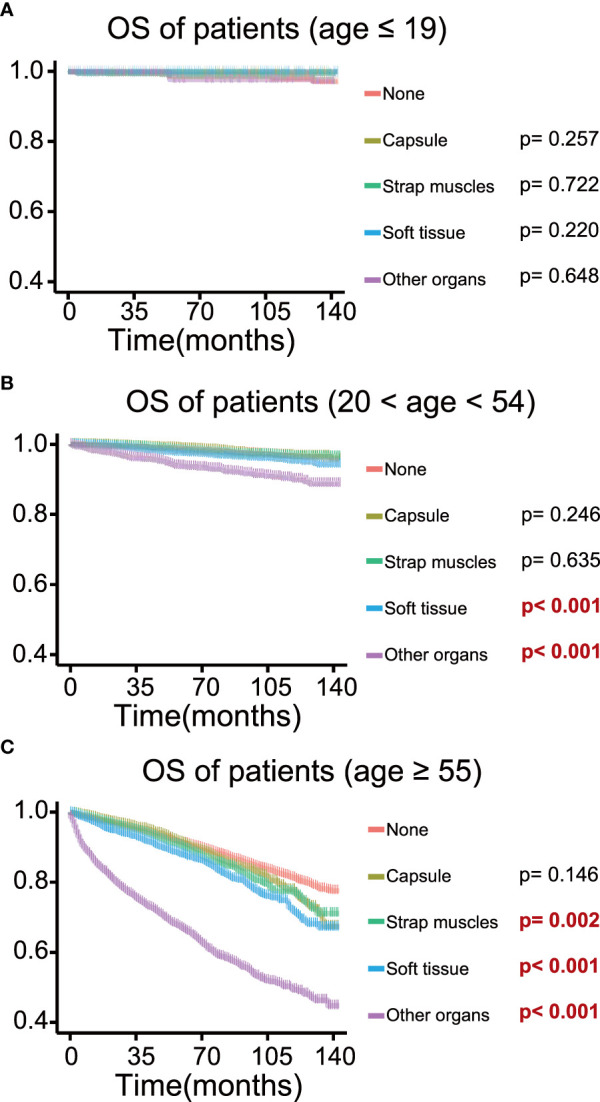
Kaplan–Meier survival curves of patients in different age groups for overall survival according to the extent of extrathyroidal extension; survival probability ranges from 0.4 to 1.0. **(A)** Age ≤ 19 group; **(B)** age 20–54 group; **(C)** age ≥ 55 group.

**Figure 5:**
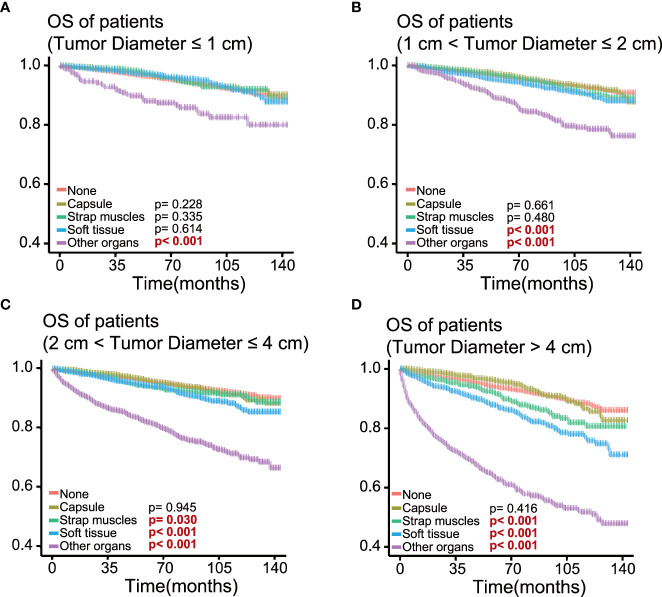
Kaplan–Meier survival curves of patients in different tumor size groups for overall survival according to the extent of extrathyroidal extension; survival probability ranges from 0.4 to 1.0. **(A)** Tumor size ≤ 1 cm group; **(B)** 1 cm< tumor size ≤ 2 cm group; **(C)** 2 cm< tumor size ≤ 4 cm group; **(D)** tumor size > 4 cm group.

To determine the effect of the exact subgroup on the prognosis of ETE and to investigate why extension into the strap muscles had an inconsistent effect, we performed a subgroup analysis of all patients with respect to all factors, in which we found that age and tumor size were important factors influencing the effect of extension into the strap muscles. In the subgroup of age ≥55 years, the OS rate of patients with ETE into or beyond the strap muscles was statistically significant (*p* = 0.002) compared with patients without ETE (**Figure 4**). In contrast, extension into the strap muscles did not affect the prognosis of younger patients. Similarly, patients with ETE into or beyond the strap muscles had a lower OS rate (*p*< 0.001) than patients without ETE when the tumor size was greater than 2 cm, as shown in **Figure 5**. Extension into soft tissues did not significantly decrease the survival time of patients with papillary thyroid microcarcinoma. In conclusion, older age (≥55) and larger tumor size (>2 cm) were mainly unfavorable factors for the OS rate of PTC with ETE into or beyond the strap muscles.

## Discussion

In observational studies focusing on risk characteristics of malignancy, Simpson’s paradox could be a common phenomenon and severely compromise the robustness of the analysis. Several methods have been developed to eliminate the influence of covariates in causal inference. We used three statistical methods (IPTW, SMRW, PSM) to process the data in order to obtain more convincing results independent of ETE effects. During PSM, loss of analytic population is inevitable. Since ETE patients tend to have more risk factors, the matched sample from the non-ETE group tends to have similar adverse clinicopathologic features. Thus, negative effects could be inferred from the comparison between matched ETE and non-ETE samples. On the contrary, IPTW and SMRW preserved all samples and details without a lack of representative characteristics of all thyroid cancer populations. Due to the calculation, IPTW gives the effect of the population average treatment effect (ATE), while SMRW presents the average treatment effect of the treated (ATT). In our analysis, we analyze the ETE in thyroid cancer instead of the treatment. The SMRW result better describes the potential adverse effect on the survival and prognosis of ETE patients. Importantly, the matching and weighting processes reduce bias from confounding factors, resulting in similar and robust results.

In our work, 23.3% (*n* = 25,302) of the patients have invasive thyroid cancer at least to the capsule. In the survival analysis before adjustment, capsular invasion is a risk factor for TCSS. After our matching and weighting process and sensitivity analysis, we clarified that capsular invasion alone could not be a prognostic marker for thyroid cancer in all subgroups.

ETE into the strap muscles was not a risk factor for OS but was still a risk factor for TCSS after adjustment. TCSS represents cancer-specific survival and better reflects the impact of thyroid cancer on individual patients. It is understandable that the small effect of strap muscles showed different effects on OS and TCSS. As shown in **Figure 4**, age was an important factor in the analysis of strap muscles. Age was also an essential element in the cause of death, which could lead to the different effects of strap muscles on OS and TCSS. Previous reports demonstrated that minimal ETE into the strap muscles only did not increase the risk of recurrence and death and was not considered a poor prognostic factor in PTC patients (24, 25, 30, 39–41). Regarding macroscopic extension into the strap muscles, Eyun Song et al. showed that tumor anterior extension into the strap muscles had no significant effect on disease-specific survival (4). Similarly, Genpeng Li et al. suggested that the extension of the strap muscles could not be considered as an independent predictor of recurrence in PTC patients (42). Based on these findings, some works have shown that low-dose radioiodine therapy is sufficient to treat patients with minimal or gross extension into the strap muscles (43–45). However, in a study of 596 PTC patients, both minimal and gross ETE into the strap muscles were independent risk factors for recurrence in PTC patients (46). The controversy about extension into the strap muscles may be due to the fact that the thyroid gland is only covered by an incomplete fibro-adipose connective tissue, which is histologically defined as a pseudo-capsule instead of a well-developed capsule (47). It could be explained by this phenomenon that capsular extension in thyroid cancer is not a well-defined concept nor has a significant impact on patient prognosis. The thyroid gland and strap muscles, sometimes soft tissues, migrate to each other and form a mixed area microscopically (48). The mixed pathological pattern brings the difficulty of consistent diagnosis of ETE (49), which may be one reason why the research studies on strap muscles present so much diversity. However, the sensitivity analysis reminded us that older age (age ≥ 55) or larger tumor size (diameter > 2 cm) all played detrimental roles in the effect of strap muscle invasion on OS. This may suggest that patients with ETE in the strap muscles as well as older age (age ≥ 55) or larger tumor size (diameter > 2 cm) need more drastic treatment.

In our analysis, invasion into perithyroidal soft tissues or adjacent organs is a risk factor for OS and TCSS before and after adjustment. In another retrospective study involving 3,267 PTC patients, soft tissue invasion was also an aggressive phenomenon, even conferring worse survival in patients than those with lymph node metastasis (49). Taking into consideration the gross invasion of the strap muscles, we believe that the gross extension into the soft tissues should also be mentioned in the staging of thyroid cancer and should be considered as a signal for aggressive treatments, including surgery and radioiodine therapy. On the other hand, research on T4a tumors has shown that extensive ETE into adjacent organs is a significant predictor of poor prognosis (50). Our work has given supportive evidence that any extension into adjacent organs could substantially impair patient prognosis, which coordinated with other research that major neck structure invasion leads to early distant and locoregional recurrence (51). Although papillary thyroid microcarcinoma (PTMC) was considered to be less aggressive, extension to adjacent organs would still worsen the clinical survival of PTMC patients in our study and also indicated in other studies (8, 52–55). On the one hand, our work confirmed the negative effect of invasion into soft tissues and other organs on patient prognosis, and on the other hand, we proposed that age and tumor size could be taken into account when thyroid tumors only invaded the strap muscles. Specialized treatments should be carried out for different patients to promote precise and individualized treatment.

This retrospective analysis also has some limitations. We filtered out 8% of all patients (9,436 out of 117,862) because of missing values. Multiple imputations could be used to retain more samples but could introduce unknown bias. In addition, recurrence data and treatments (thyroid-stimulating hormone suppression or radioiodine therapy) were not analyzed in this work, so we are unable to report locoregional or distant recurrence or the effect of other treatments.

## Conclusion

This is a retrospective study using data from the SEER database to evaluate the effect of different extents of ETE on survival in PTC patients. According to our results, ETE in soft tissues or other organs is a high-risk factor in papillary thyroid cancer. As for PTC patients with ETE in the strap muscles, older age (≥55 years old) and larger tumor size (>2 cm) are of great importance in impaired prognosis and, thus, should be taken into consideration in the treatment option.

## Data availability statement

The original contributions presented in the study are included in the article/**Supplementary Material**. Further inquiries can be directed to the corresponding authors.

## Author contributions

MX: conceptualization, validation, formal analysis, data curation, and writing—original draft. ZX: conceptualization, visualization, data curation, and writing—original draft. QZ: investigation and writing—original draft. WY: formal analysis and data curation. PY: formal analysis and writing—review and editing. JT: methodology and visualization. JZ: validation, methodology, supervision, and writing—review and editing. TH: conceptualization, methodology, supervision, and writing—review and editing. All authors contributed to the article and approved the submitted version.

## Supplementary material

Supplementary Figure 1 Standardized mean differences (SMD) comparison of propensity score matching (PSM), inverse probability of treatment weighting (IPTW) and standardized mortality ratio weighting (SMRW).
