# Supplementary figures and images for: Causal inference between aggressive extrathyroidal extension and survival in papillary thyroid cancer: a propensity score matching and weighting analysis
Source: Front Endocrinol (Lausanne). 2023 May 24;14:1149826. doi: 10.3389/fendo.2023.1149826 (PMC10244725; doi:10.3389/fendo.2023.1149826)

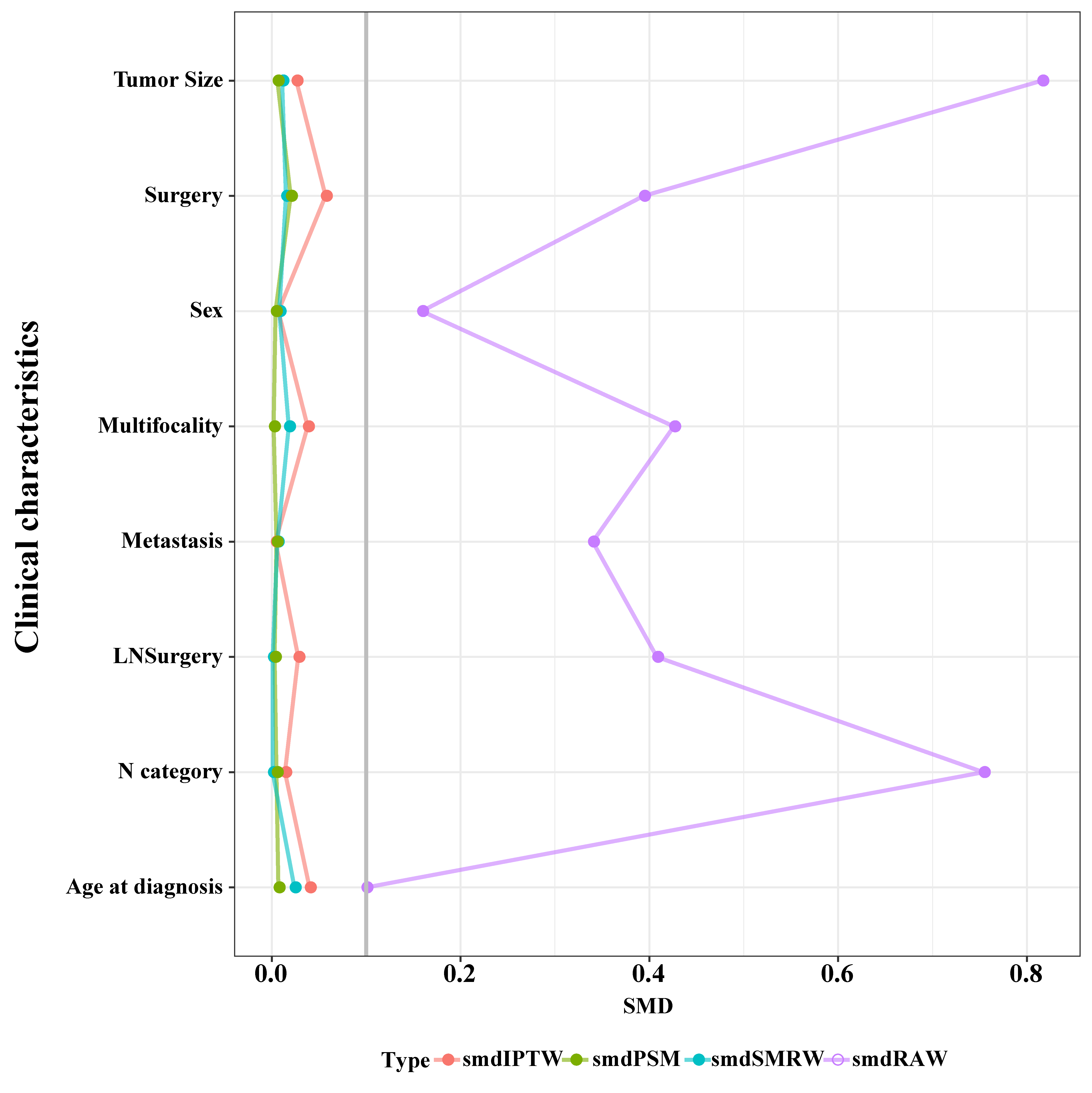

Supplement: Supplementary Figure 1 — Standardized mean differences (SMD) comparison of propensity score matching (PSM), inverse probability of treatment weighting (IPTW) and standardized mortality ratio weighting (SMRW). [file Image_1.tif]
